# Animal models in medical translation: the grand challenge of developing new treatments for human diseases

**DOI:** 10.3389/fmedt.2024.1367521

**Published:** 2024-02-16

**Authors:** Philip V. Peplow

**Affiliations:** Department of Anatomy, University of Otago, Dunedin, New Zealand

**Keywords:** animal model, medical translation, new treatments, human, diseases

## Introduction

Animal models have been developed for many human diseases including cardiovascular, respiratory, hepatic, renal, ophthalmic, metabolic, neurologic, neurodegenerative, neuropsychiatric, inflammatory, and infectious diseases or conditions. Their use has proved crucial in developing treatments for a large number of human diseases and testing implantable devices. While a single animal model may not show all the main pathophysiological changes in different human diseases, they are the most valuable tool for studying treatment strategies prior to performing clinical trials. Cell culture and molecular biology studies are used to support the findings from the use of animal models. Selective breeding, genetic modification, and advances in molecular imaging provide a better understanding of disease processes and insights into possible new interventions. There are several areas in which future research using animal models could make an important contribution to understanding disease processes and possible new treatment strategies.

### Use of nanoparticles

Incorporation of therapeutic molecules into nanoparticles may be suitable for the treatment of neurodegenerative diseases and brain injury (1). Animal models of Alzheimer's disease, Parkinson's disease, Huntington's disease, ischemic stroke, amyotrophic lateral sclerosis, traumatic brain injury, multiple sclerosis, epilepsy have been developed (2–8) and could be used to test the effect of administering nanoparticles intravascularly or directly into the brain. The nanoparticles could be loaded with therapeutic agents such as neurotrophins (e.g., neurotrophin 3, brain-derived neurotrophic factor) and growth factors (9), cerium oxide or made of graphene (10), or be used as magneto-electric nanoparticles (11). The latter can be subjected to ac-magnetic field stimulation and cause stimulation of neurons in regions of the brain that the nanoparticles are guided to. It has the potential for deep brain stimulation in animal models of Parkinson's disease. In neurodegenerative diseases, passage of nanoparticles through the blood-brain barrier is facilitated due to damage occurring to the blood-brain barrier (12, 13). Nanoparticles have been used for the treatment of cancer (14, 15), liver fibrosis (16), and diabetes (17). Mouse, rat, and monkey models have been developed for many neurodegenerative diseases, and for example magnetically guided delivery of magneto-electric nanoparticles was tested in the brains of mice and their distribution to different cell types (11).

### Use of bioactive scaffolds

Incorporation of therapeutic molecules into mini scaffolds to form bioactive scaffolds can be used for the treatment of neurodegenerative diseases, multiple sclerosis, epilepsy, traumatic brain injury, stroke (18), skin regeneration (19), cartilage/bone repair (20–22), cardiac repair (23), and soft tissue repair (24). Metal nanoparticles-based scaffolds have been used for bone tissue regeneration (25). Studies on skin regeneration may be performed using small or large laboratory animals (e.g., mouse, piglet), while cartilage/bone repair can be examined using large laboratory animals (e.g., dog, sheep). In a recent review of biomaterial and tissue engineering strategies for the treatment of brain neurodegeneration (26), a wide variety of biomaterials had been used including nanoparticles, carbon nanotubes for cell engraftment, microspheres and microscale scaffolds, functionalized composite scaffolds, self-assembling peptides as scaffolds, and micro-tissue engineered neural constructs, and were tested in mouse and rat models. *In vitro* studies involving measurement of neural aggregate + axon length, and *in vivo* studies examining neurorestorative effects of biomaterial and tissue-engineered constructs using animal models of traumatic brain injury or Parkinson's disease have been reported. Alginate fibres have recently received attention as a possible treatment modality of amyotrophic lateral sclerosis. Alginate fibres cross-linked with strontium and loaded with methylene blue can enhance the survival of motor neurons (27). Also, *in vitro* models of traumatic brain injury can be used to test therapeutic materials. Monocultures of cortical neurons can be established, and an injury created using a pipette tip or needle. Complex *in vitro* systems have been developed such as a mixed glial/polyglial culture system in which astrocytes, oligodendrocytes and microglia are present in reproducible ratios. This model is suited to studying glial responses to therapeutic materials. The model has evolved to include the neuronal population alongside multiple glial cells and has been used to study delivery of nanoparticles to the injury site (28).

### Use of liposomes

Liposomes are nanosized vesicles consisting of a phospholipid bilayer membrane enclosing an aqueous compartment. The structure of the lipid bilayer membrane enables liposomes to immobilize both hydrophilic drugs in their aqueous core and hydrophobic drugs within the lipid bilayer, and they have great potential as smart drug delivery systems (SDDSs). They are highly biocompatible, biodegradable, and non-toxic to the body. In addition, they have high drug loading capacity and high solubility in water and blood. Incorporation of chemotherapeutic drugs into liposomes that can be injected into the vascular system and can be released upon breakdown of the liposomes by ultrasound/laser irradiation (29–31) has been used in treating tumors, and several successful liposomal formulations for cancer treatment are currently available (30). Upon injection into the bloodstream, serum proteins (called opsonin proteins) bind to the surfaces of liposomes, making them susceptible to phagocytic attack and removing them from the blood circulation and lowering their accumulation at targeted diseased sites. By coating liposomes with hydrophilic molecules such as polyethylene glycol, the adsorption of opsonin proteins on their surface is reduced, thus protecting them from phagocytic attack, increasing their circulation times to more than a day, delivering the entrapped chemotherapeutics at the targeted tumor sites, and decreasing cytotoxic effects on normal cells. Liposomes carrying therapeutic drugs have the potential to treat heart conditions (e.g., angina, coronary artery disease caused by atherosclerosis), kidney disease, and liver disease. Liposomes can cross the blood-brain barrier using receptor-mediated transcytosis (32) and could be used to treat neurodegenerative diseases (e.g., Alzheimer's disease, Parkinson's disease) (33) for which animal models have been developed (2–8). Liposome nanoparticles conjugated with lactoferrin to deliver neuronal growth factors across the blood-brain barrier had a protective effect against amyloid beta-induced neurotoxicity *in vitro* (34). A magnetic (Fe_3_O_4_-nimodipine) liposomal delivery system was developed by modifying nimodipine with polyethylene glycol-coated Fe_3_O_4._ In a rat model of Parkinson's disease, enhanced protection of dopaminergic neurons was observed by reducing the neurotoxicity through nimodipine incorporated in liposomes (35).

### Use of stimuli-responsive carriers

Many important food bioactive compounds have applications in health promotion and disease prevention. However, these compounds have low chemical stability and bioavailability. Recently there has been a major research effort to develop advanced delivery systems of natural bioactive molecules. Stimuli-responsive carriers have potential for improving delivery and release of intact bioactive phytochemicals to target sites in response to certain stimuli or combinations of them (e.g., pH, temperature, oxidant, enzyme, irradiation), thereby increasing therapeutic outcomes and reducing side effects (36). Hybrid formulations (e.g., organic-inorganic complexes) and multi-stimuli responsive formulations have been investigated for smart-delivery of food bioactive compounds such as quercetin, curcumin, resveratrol. In the extracellular tissues of many solid tumors the pH is around 6.5 while in healthy tissues it is 7.4–7.5. The use of certain polymers whose conformation or solubility properties are altered under particular pH conditions would result in fast nutrachemical release at a specific site. In these carriers, the pH-sensitive polymers with functional groups (e.g., carboxylic acids, amines) can act as proton donors or acceptors in response to changes in environmental pH. Protonation of polymers in acidic conditions causes structural deformation and alteration in hydrophobicity of the polymers, thereby enhancing the release of the encapsulated compounds. Other approaches involve the application of acid-labile linkages or polymers, ionizable chemical groups, and gas-generating precursors (37). A possible application could be to deliver pH-sensitive polymers to the stomach to treat gastric disease. Stimuli-responsive nanogels or hydrogel nanoparticles have application in cancer therapy, delivery of antiviral drugs, delivery of vaccines, and treatment of diabetes (38). Such systems recognize either internal physiological cues (e.g., pH, temperature, redox e.g., glutathione) (36) or respond to externally applied stimuli (e.g., temperature, magnetic fields, photons, ultrasound waves). These stimuli-responsive nanogels have an internal hydrophilic nature for drug and biomolecule encapsulation, enhanced stability for blood circulation, and controlled release of the loaded drug or biomolecule.

### Use of colon-targeted drug delivery systems (smart pellets)

Colonic drug delivery systems have been used to treat intestinal diseases such as colorectal carcinoma, ulcerative colitis, diverticulitis, Crohn's disease, and irritable bowel syndrome. By reducing unwanted adsorption in other regions of the gastrointestinal tract and ensuring that the whole drug dose is specifically delivered to the colon, colon-specific drug delivery improves therapeutic effectiveness. Most colon-targeted drug delivery systems are either responsive to the pH of the colon or to enzymes produced by intestinal microbiota. Smart pellets have been developed for controlled delivery of drugs to the gastrointestinal tract, e.g., 5-fluorouracil to treat colorectal carcinoma. Polymer-based formulations were based on hydroxyethyl methacrylate copolymerized with methacrylic acid. The system was optimized to deliver 5-fluorouracil to the colon by preventing/delaying the release of 5-fluorouracil within the first 5–6 h following oral administration to ensure drug arrival to the colonic region. This enhances therapeutic outcome, reduces dosing and undesirable side effects, and increases patient compliance. Six drug-loaded formulations were produced with a drug entrapment efficiency of approximately 91% in the formulations. Less than 27% total drug release occurred for all formulations after 5 h in the *in vitro* release study, and the highest total release after 24 h was 69% (39). *In vivo* studies of smart pellets for administering drugs to the colon have been performed in rats and rabbits.

### Use of smart pills and ingestible sensors

Smart pills can be used to monitor patients with chronic diseases such as heart disease, gastrointestinal disorders, and diabetes, during surgeries, in critical care settings, or in studying physical responses during physical activities such as sports performance (40). Smart pills can also be used for drug delivery. A microchip sensor in the pill monitors the effectiveness of the drug and alters the dosage to ensure the optimal amount is taken for the condition being treated. This can decrease side effects and improve treatment outcomes. Smart pills have the potential to reduce healthcare costs (41). Ingestible sensors or smart pills have been developed for the imaging of esophagus/stomach/small intestine (as a gastrointestinal tract diagnostic tool), sensing different types of gases to provide metabolic and digestive information, monitoring medication compliance or absorption of medication (e.g., in schizophrenia patients), and electrochemical signal sensing (on stools as a gastrointestinal tract diagnostic tool) (42, 43). Animal models that have been used for studying gastrointestinal disease include mice, rats, guinea-pigs, dogs, pigs (44–46).

This Speciality Grand Challenge is a personal opinion of some of the new treatment modalities that can be explored using animal models of disease. More details on the applications are available in the cited references and may include the animal models that have been used. Other important challenges are included in the mission and scope of this section, which aims through the use of animal models to improve patient health outcomes.
